# Study protocol: The development of a pilot study employing a randomised controlled design to investigate the feasibility and effects of a peer support program following discharge from a specialist first-episode psychosis treatment centre

**DOI:** 10.1186/1471-244X-10-37

**Published:** 2010-05-25

**Authors:** Jo Robinson, Annie Bruxner, Susy Harrigan, Sarah Bendall, Eoin Killackey, Vittoria Tonin, Katherine Monson, Melissa Thurley, Shona Francey, Alison R Yung

**Affiliations:** 1 Orygen Youth Health Research Centre, Centre for Youth Mental Health, Department of Psychiatry, University of Melbourne, Locked Bag 10, 35 Poplar Road, Parkville, Victoria 3052, Australia

## Abstract

**Background:**

Young people with first-episode psychosis (FEP) are at risk of a range of negative outcomes. Specialist FEP services have been developed to provide comprehensive, multi-disciplinary treatment. However, these services are often available for a restricted period and the services that young people may be transferred to are less comprehensive. This represents a risk of drop out from treatment services in a group already considered to be at risk of disengagement. Peer support groups have been shown to improve social relationships among people with psychosis however individual peer support programs have not been tested on young people with first-episode psychosis; nor have they been tested at the point of discharge from services.

**Methods/design:**

The study is an 18-month randomised controlled trial being conducted at Orygen Youth Health Research Centre in Melbourne, Australia. The aim of the study is to test the feasibility and effects of a 6-month peer support intervention delivered to young people with FEP over the period of discharge. Participants are young people aged 15-24 who are being discharged from a specialist first-episode psychosis treatment centre. There is a 6-month recruitment period. The intervention comprises two hours of contact per fortnight during which peer support workers can assist participants to engage with their new services, or other social and community activities. Participants will be assessed at baseline and post intervention (6 months).

**Discussion:**

This paper describes the development of a randomised-controlled trial which aims to pilot a peer support program among young people who are being discharged from a specialist FEP treatment centre. If effective, the intervention could lead to benefits not only for participants over the discharge period, but for peer support workers as well.

**Trial registration:**

The study was registered with the Australian New Zealand Clinical Trials Registry; number: ACTRN12610000241033.

## Background

Young people with first-episode psychosis (FEP) are at risk of a range of negative outcomes both in clinical terms and with regard to their general and social and vocational functioning. Specialist FEP services have been developed to provide comprehensive, multi-disciplinary treatment of the medical, social, psychological, family, educational and vocational aspects of the experience of FEP, in order to help young people to recover from psychosis. However, these services are often available for a restricted period and the services that young people may be transferred on to are less comprehensive. This represents a risk of drop out from treatment services in a group already considered to be at risk of disengagement from services [1]. A goal of a FEP service should be to manage the transition to a new service to ensure good engagement with the new service, which it is hoped would lead to improved treatment outcomes.

Individuals with FEP are also at high risk of suicide and suicide attempt (SA). Rates of suicide are estimated at between 1 and 3% over a 4 to 5-year follow-up period [2,3]. Rates of deliberate self-harm (DSH) and SA are higher; between 10% and 14% of people with FEP report either DSH or a SA prior to presentation for treatment [4,5,2]. Rates remain high following the commencement of treatment. One-year prevalence rates of SA range from 2.9% to 11% [4,6,7]. Longer term follow-up studies have reported a 2-year prevalence rate of 11.3% [8] and a 4-year prevalence rate of 18.2% [2].

Evidence also tells us that the strongest predictor of future suicide and SA is a previous attempt [9,3]. Other key risk factors include depressive symptoms and recent discharge from hospital [7,10-13]. One way to reduce the risk of suicide at discharge is to provide an enhanced support system for the young person throughout the discharge process and peer support may be ideal in this area.

The concept of peer support is based on the notion that people who have experienced and overcome a particular type of adversity can be a useful source of support, encouragement and hope to others experiencing similar situations [14]. A survey of consumer perspectives on the management of psychiatric emergencies identified peer support as a future priority for use in emergency settings [15]. A further study tested the effect of peer support groups on people with psychosis [16] and although this lead to an improvement in the social relationships of participants, it was limited in several ways. Firstly, it was not tested specifically with young people with FEP. Secondly, it was not conducted at the point of discharge. Thirdly, the authors did not look at suicidality as an outcome of interest, and fourthly, it was a group and not an individual intervention.

It has been concluded that whilst peer support appears to have become a popular addition to mental health services and has the potential to be an important component of mental health care, it is to date under-researched [14]. Further, to our knowledge, it is not yet known what the essential components would be when delivering a peer support program to young people with FEP over the period of discharge from a specialist service. Nor do we know if it is feasible to expect young people who are in recovery themselves to support other young people at this vulnerable time.

## Methods/design

### Aims and hypotheses

The overall aims of this study are to test the feasibility and effects of a 6-month peer support intervention delivered to young people with FEP from approximately 3 months prior to discharge from a specialist treatment service, and continuing for 3 months post-discharge. We also aim to identify what participants want from a peer support program at this time. If the program proves effective and acceptable to participants, we aim to develop a treatment manual to support future programs.

The hypotheses are that when compared to treatment as usual (TAU) the peer support intervention will lead to: 1. Increased engagement and treatment adherence among people being discharged from the Early Psychosis Prevention Intervention Centre (EPPIC) and who are being referred on to alternative services; 2. Increased perceived social support; 3. Increased quantity and improved quality of information provided; 4. Increased service satisfaction; 5. Reduced risk of suicide; and 6. High levels of satisfaction with peer support role for recipients and peer support workers.

### Study design

The study is a randomised controlled trial registered with the Australian New Zealand Clinical Trials Registry and approved by the North Western Mental Health Research and Ethics Committee.

### Setting

Orygen Youth Health Research Centre (OYHRC) is a publicly funded specialist mental health service for people aged 15-24 living in the Western and North western regions of Melbourne with an integrated research centre. The service treats individuals with both psychotic and non-psychotic disorders. There is also a youth participation program which comprises consumer advocacy, consultation, public speaking, a drop in room and a peer support program. The peer support program currently offers peer support to young people on the inpatient unit. This study will see an extension of the peer support program to include the period leading up to and immediately following discharge from the service. The current study will take place in EPPIC and represents a partnership between EPPIC, the Orygen research centre and the youth participation program.

### Participants

**Figure 1:**
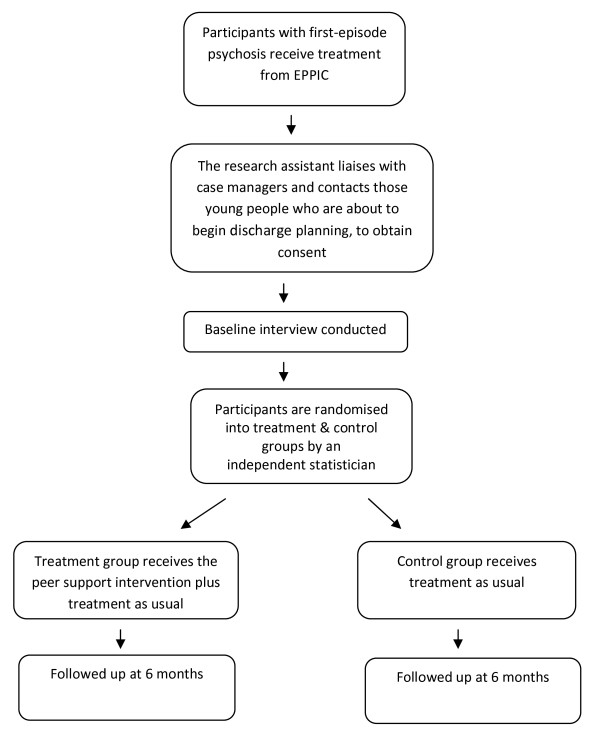
**Study flow-chart: This figure displays the participant flow-chart**.

Participants will be approximately 36 young people, aged 15-24, who are being discharged from EPPIC. There are currently around 300 young people registered with EPPIC, between 10 and 20 of whom are discharged per month. For the purpose of the study protocol we have assumed that on average 12 people will be discharged per month and that 50% (n = 6) will consent to participate. This would mean that 3 people would be recruited to the treatment group and 3 people would be recruited to the control group per month. The study has a 6-month recruitment period which would give us a sample of approximately 36, 18 in the treatment and 18 in the control group. It is anticipated that the sample would be reduced by follow-up attrition, resulting in an approximate final study sample of 28-31 subjects. Please see Figure 1.

### Procedure

The study team comprises researchers and representatives from the clinical program. The research assistant (RA) will attend the regular clinical meetings held at EPPIC in order to identify young people who are about to commence the discharge planning period. Once the discharge planning process is about to begin the RA will contact the young person and see if they are interested in the study. If so they will obtain written consent and conduct the baseline assessment (see below). They will also provide written information for the participant to keep, which explains the nature of the intervention and provides contact details for the research team.

Once the baseline assessment is complete the young person's details will be passed to an independent statistician (SH) who will randomise them into either the treatment or control group using randomly permuted blocks of varying size, to help ensure that subject allocation to the treatment groups is approximately equal. Once randomisation has taken place the statistician will contact the project coordinator (JR) who will provide the relevant details to the peer support coordinator (MT/VT) in order that the intervention can begin. The peer support coordinator will then allocate a peer support worker (PSW). The first meeting between the PSW and the participant will take place on-site with the case manager and the peer support coordinator present. The nature of the intervention will be explained again and both the participant and PSW will sign a prepared document to indicate that they fully understand the terms of the intervention.

### Intervention

Those participants randomised to the treatment group will receive treatment as usual plus the peer support intervention. The intervention will be delivered by the peer support workers from the point that discharge planning commences until 3 months after discharge from the service. It is anticipated that this will be around 6 months in most cases. The intervention will adopt a befriending model and will comprise an in-reach and out-reach service whereby participants will have a nominated PSW who they can visit at the drop-in centre at OYH or who they can meet off-site for support sessions. The PSW will accompany young people to GP or other health appointments, help them to locate information about mental health services and social groups, and help them find ways to ensure good mental health once the program is over. A total of 2 hours contact per fortnight of contact will be offered.

A phased intervention approach will be adopted whereby contact in the initial and final months will be minimal compared to the time around discharge. During the first two months (initial phase) the PSW will work on building rapport, developing and maintaining contact, and assisting the young person with mental health issues and service information. It is expected that most contact will be in-service as the young person will still be attending EPPIC. This will allow for more face-to-face contact which is important to the development of a 'buddy' relationship. Nearing the discharge period and shortly after (mid-phase) it is expected that the PSW will assist the young person in their transition to a new service. PSWs will be available to escort the young person to the new service, assist them with attending sessions, understanding health services (including the broader mental health service), and motivating the young person to consider supports and contacts for the future. This period of the intervention will be the most intensive and important for ensuring treatment engagement and harm minimisation. The PSW and young person will negotiate when and how to allocate their 2 hours per fortnight and it is anticipated that while most time will be spent face-to-face, a small proportion will also be allocated to phone and email contact. The final phase of the intervention will involve the PSW 'weaning' the young person from the support of the PSW by encouraging the young person to be proactive in managing their mental health. Unlike the initial phase, most contact in the final stage will be conducted via phone or email and will focus on encouraging the young person to develop other new (preferably social) contacts and supports. The young person will be reminded that at the cessation of the study they will no longer be able to rely on the PSW for support.

One of the aims of the study is to identify what young people actually want from a peer support program therefore the protocol will be necessarily flexible in order that the intervention can meet the individual needs of the participants. The PSW will be required to record all contacts in a log book and this information together with participant and PSW feedback will inform the development of an intervention manual for future use.

### Control intervention

Those in the control group will receive TAU.

### Outcomes

The primary outcomes of interest are whether or not the intervention leads to better levels of engagement and treatment adherence, higher levels of perceived social support, a greater quantity and quality of service related information received and greater service satisfaction among the treatment group than the control group. An additional outcome of interest is whether or not the intervention leads to reduced suicide risk, defined as the presence of current or recent suicidal ideation and/or suicidal behaviour including deliberate self-harm. Because depression is highly correlated with suicide risk this will also be measured. These will be assessed via a face-to-face interview conducted by the RA at baseline (i.e. prior to commencement of the intervention) and will be repeated post-intervention (i.e. 6 months later).

Process evaluation questionnaires have been specifically developed to evaluate the above mentioned themes. These brief questionnaires will be administered monthly via face-to-face or telephone interviews with control and intervention group participants and PSWs. The three questionnaires (one for each group) each contain mirrored questions to enable cross comparisons between groups. For example, those in the intervention group are asked how much having a PSW increased their motivation to continue with their mental health treatment while the PSW is asked to rate how much they think they increased each participant's motivation. Similarly, the control group is asked how much more motivated they expect they would have been, had they had a PSW. The questionnaires each contain qualitative and quantitative items; the quantitative items will be analysed at the cessation of the study to evaluate the intervention during each of the phases while the qualitative items will be used to direct the intervention throughout the study. For example, if PSWs make suggestions for more training or support then the intervention will be amended accordingly. Qualitative data and amendments will also be evaluated at the cessation of the study.

### Outcome measures

Basic demographic details, including age, gender, employment or educational status, living circumstances, country of birth, parents' or guardians' employment status, parents' or guardians' country of birth, medical history and details of any treatment being received, are recorded on a specifically designed, standardised questionnaire.

Perceived social support will be measured by the Multi-dimensional Scale of Perceived Social Support [17]. It is a 12 item measure using a 7 point likert scale which assesses perceived social support in family, friends, and social others.

Service satisfaction will be measured using the Verona Service Satisfaction Scale [18]. Participants are asked to rate this 32 item questionnaire and complete two qualitative questions regarding likes and dislikes about the treating service.

Engagement and treatment adherence will be measured by The Psychosocial Treatment Compliance Scale [19], by specific questions included in a treatment questionnaire (see below) and via a telephone call made by the RA to the new treating clinician asking about attendance at appointments and medication adherence. These treatment adherence measures were created specifically for the current project and assess medication and session adherence.

Quality of life will be measured using the Heinrichs Quality of Life Scale [20]. This 21 item questionnaire has numerous prompts enabling a comprehensive understanding of the persons' life. All items will be administered and rated by the RA.

The Reynolds Suicidal Ideation Questionnaire is a 25 item self-report measure assessing suicidal ideation in the past month [21]. Each item will be scored on a 7 point likert scale of frequency ranging from "almost every day" to "I never had this thought".

The Suicide Behaviours Questionnaire (SBQ) [Linehan, M. M. Suicide Behaviours Questionnaire. *Unpublished instrument*] measures five domains: past suicidal ideation, future suicidal ideation, past suicide threats, future suicide attempts, and the likelihood of dying in a future suicide attempt which are rated across a number of time points: (1) the past several days including today, (2) past month, (3) past four months, (4) past year, and (5) lifetime. The SBQ-14 also assesses the number of past episodes of DSH.

The Beck Depression Inventory [22] is a multiple choice self report measure used to assess the severity of depression.

The follow-up questionnaire will also include a series of evaluation questions designed to elicit whether or not participants liked the intervention and whether or not they found it helpful. The questionnaire will also ask about which aspects of the intervention participants found most helpful, which were least helpful and what if anything they would have liked to have been included in the intervention that was not provided. Those in the control group will be asked to identify what support they think they would have liked to receive from a PSW over the discharge period. This questionnaire will also assess participants views of the quality and quantity of relevant information received at the point of discharge. It will be administered at the end of the interview in order that the RA can remind blind to treatment throughout the majority of the assessment.

### Sample size calculation: effect size and statistical power

The proposed sample size of 36 subjects is anticipated to be further reduced by follow-up attrition, resulting in an approximate final study sample of 28-31 subjects. Based on the proposed sample size, the study is sufficiently powered (80%) to detect only very large effects (*d *= 1.0 using Cohen's terminology [23]), assuming a 2-tailed test with alpha at 0.05. Given the reduced power of the study to detect smaller effects than this, inferential statistics will play a relatively limited role in this pilot study, with the focus primarily descriptive in nature.

### Randomisation/treatment allocation

Random allocation to the treatment or control group will be carried out by an independent statistician (SH) who has no knowledge about participants' characteristics, using randomly permuted blocks of varying size, to help ensure that subject allocation to the treatment groups is approximately equal. This will be concealed from the research team. The statistician will notify the study coordinator regarding the group allocation.

The RA, who carries out the assessments, will be blind to group allocation. The success of blinding will be assessed at the follow-up assessment via questioning the RA whether they think the participant received peer support as part of the RCT - Yes/No.

### Statistical methods

A range of descriptive statistics will be used to compare the groups on key outcome measures at follow-up, including level of engagement and treatment adherence, perceived social support and service satisfaction. Additionally, symptom and outcome measures will be assessed, including suicidal ideation/behaviour, severity of depression and functional outcome. The extent of any baseline differences on these measures will also be examined and taken into account if necessary. Furthermore, identification of the key components of an effective peer support program based on the perspective of the client will provide vital qualitative information which will be used to design future programs.

### Safety and supervision

All PSWs will receive the same training prior to commencing the program. This will comprise an introduction into peer support work, information regarding local services, training in managing clinical aggression, Mental Health First Aid Training [24] and suicide risk management training (Applied Suicide Intervention Skills Training (ASIST)) [25]. In addition a treatment protocol will be prepared by the PSW supervisor and the research team which will clearly set out what the intervention will and will not include, and what the PSW is and is not expected to do. Fortnightly supervision sessions will be provided by the supervisor for all PSWs. These sessions will be used to provide basic supervision but also to monitor the nature of the PSW contacts and to identify any ongoing training needs.

In addition an on-call system will operate staffed by the PSW supervisor and members of the research and clinical team. All PSWs will be required to ensure that the on-call supervisor knows of the time and location of their visit. The PSW will be required to SMS the supervisor when the visit is complete. If there were any problems or concerns they will be required to telephone the on-call worker and these will be dealt with appropriately.

Regular team meetings will be held in order that any difficulties can be dealt with promptly and to facilitate close liaison between the peer support workers, the clinical representatives and the research team. This will also allow for ongoing discussion about the nature of the PSW contacts with participants. It will also ensure that the research team are fully aware when discharge planning commences and the exact point of discharge for each participant and when the intervention commences and finishes. Similarly this will enable the research team to inform the peer support workers once the baseline assessment is complete and the intervention can begin.

## Discussion

This paper describes the development of a randomised-controlled trial which aims to pilot a peer support program among young people who are being discharged from a specialist FEP treatment centre. Whilst intuitively attractive, there is little evidence regarding the potential benefits of peer support for young people with FEP currently available at this time. There is also little evidence regarding what young people want from a service such as this, and whether or not there are potential benefits for the peer support workers as well as the study participants. Thus, in addition to the primary outcomes of interest, it is hoped that this pilot study will provide some indication as to what kinds of support young people require at this time and whether or not it is realistic to expect other young people to provide this.

Through participation in the program all peer support workers will receive training and experience that will be of benefit to them once the study is complete. In addition, protocols and a treatment manual will have been developed that will be sustainable once the research is complete. However if the research indicates that the program is of benefit the study team will seek continued funding in order that the program can continue and be further tested with a larger sample.

## Competing interests

The authors declare that they have no competing interests.

## Authors' contributions

All authors have been involved in conception of the study and made substantial contributions to the study design and the development of the intervention and the study protocols. All authors have been involved in drafting the manuscript and have seen and approved the final version.

## Pre-publication history

The pre-publication history for this paper can be accessed here:

http://www.biomedcentral.com/1471-244X/10/37/prepub
